# Transjugular retrieval of a knotted peripherally inserted central venous catheter (Epicutaneo-Cava catheter) in a neonate

**DOI:** 10.1259/bjrcr.20150327

**Published:** 2016-07-28

**Authors:** Lindsay Zhou, Mathievathaniy Muthucumaru, Kenneth Tan, Kenneth Lau

**Affiliations:** ^1^Department of Paediatric Surgery, Monash Medical Centre, Melbourne, VIC, Australia; ^2^Department of Neonatology, Monash Medical Centre, Melbourne, VIC, Australia; ^3^Department of Diagnostic Imaging, Monash Medical Centre, Melbourne, VIC, Australia

## Abstract

Knotting of intravascular catheters has been well described, and all such cases documented in the literature have occurred during catheter insertion. Knot formation has not been reported during the removal of a peripherally inserted central venous line (Epicutaneo-Cava 2 French 24 gauge) in a neonate. The mechanism of knotting in our case is not fully understood. This case emphasizes the value of plain radiography in detecting the presence of a knot in the line, and is being presented to raise the awareness about knot formation if undue resistance is felt during line removal. Early recognition of this rare but serious complication may avoid line fracture and potential fragment embolization.

## Background

Peripherally inserted central venous catheters are commonly used in neonatal practice for delivery of parenteral nutrition, medications and other i.v. solutions. This mode of cannulation avoids risks involved in direct surgical/percutaneous access of the central veins in very sick and often premature neonates.^1^ The recommended sites for insertion include the basilic, cephalic, long saphenous and scalp veins.

Common complications associated with these catheters are infection, line migration and thrombus formation in the veins.^1^ Here, we report a case of an unexpected complication of knot formation that occurred during the removal of the peripherally inserted central venous catheter (PICC) in a neonate. Although knot formation in PICC has been previously reported in adults,^2^ there has been no literature documenting this in a neonate.

## Case report

**Figure 1.**
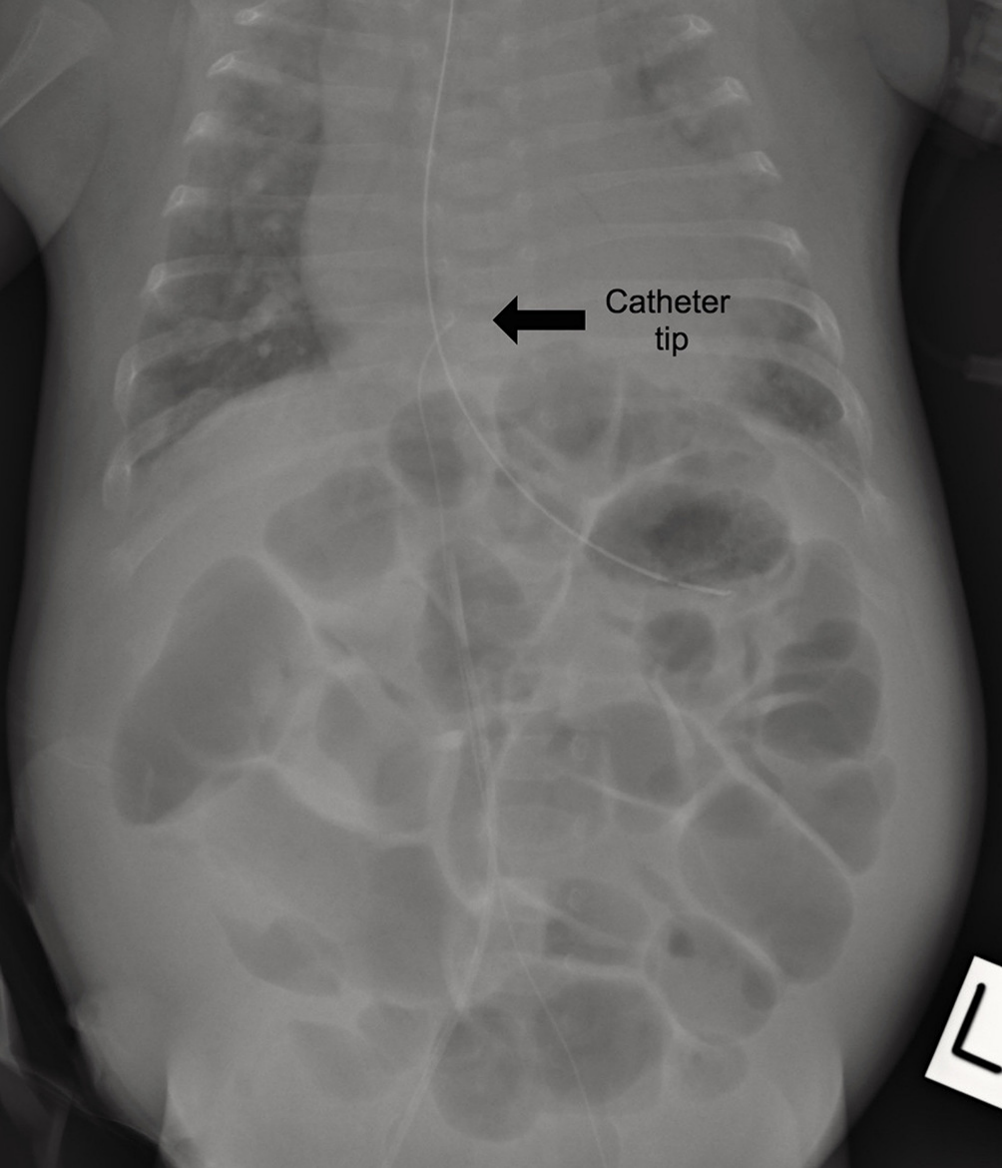
Initial chest and abdominal plain radiograph showing the tip of the PICC at the T9 level (arrow).

A female infant was delivered *via* emergency caesarean section at 29 weeks gestation weighing 1222 g (50th centile). She required respiratory support for respiratory distress syndrome for an extended period of time. In addition to other issues of prematurity, feed intolerance and abdominal distension were major ongoing challenges in her clinical management. She eventually underwent a defunctioning ileostomy owing to presumed pseudo-obstruction with no identifiable cause. A PICC [2 French 24 gauge silastic catheter (Epicutaneo-Cava catheter, Vygon, Ecouen, France)] was inserted on day 113 of life for essential i.v. access to replace a previously inserted right femoral catheter. A PICC measuring 25 cm was inserted into the long saphenous vein at the level of the left mid-calf. Initial chest and abdominal X-ray showed that the tip of the line was at T9 level with no coiling (Figure 1).

**Figure 2.**
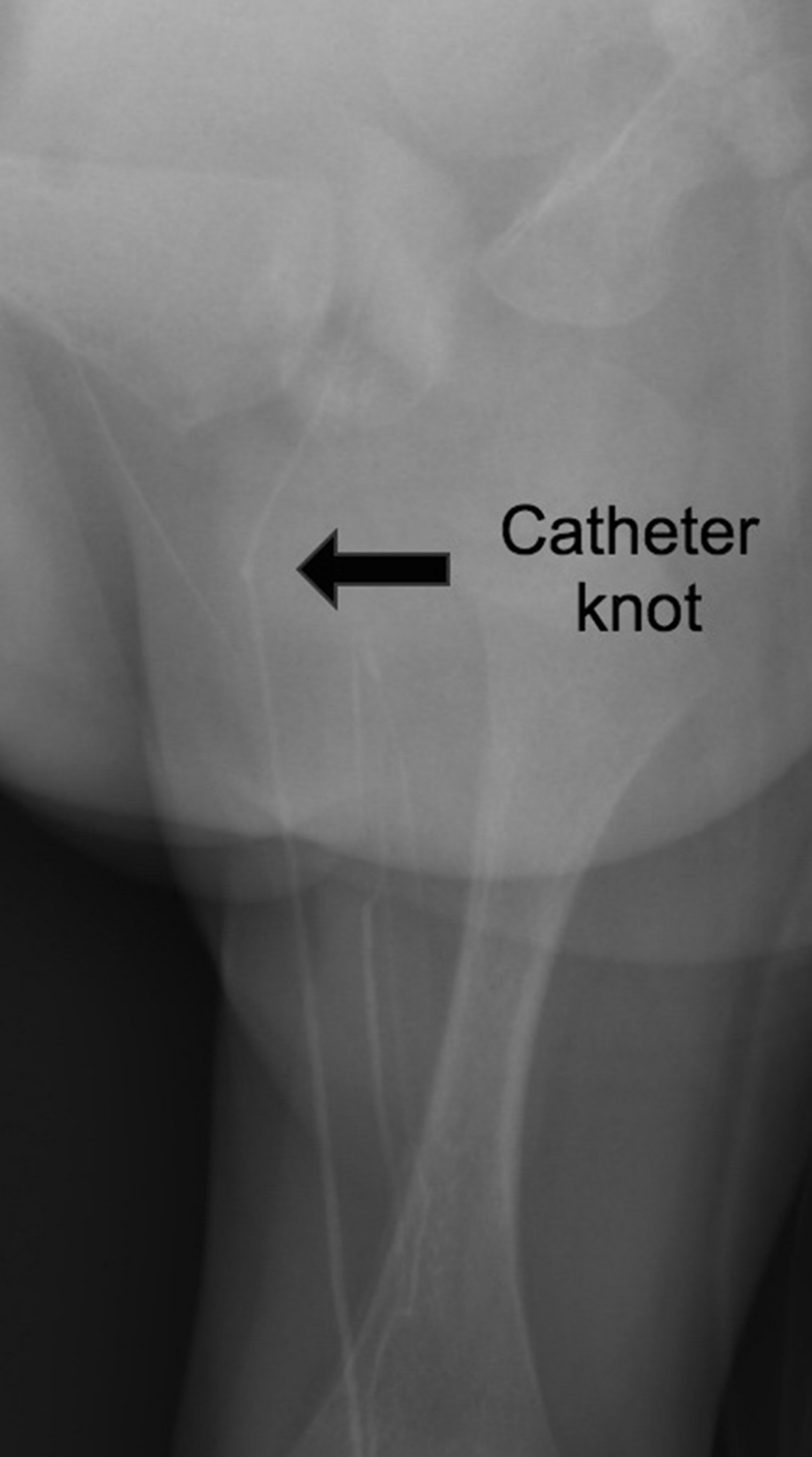
Dedicated oblique view of the left groin confirmed that a knot had formed at the distal peripherally inserted central venous catheter (arrow), preventing its removal through the calf entry site. The linear opacities projecting over the soft tissues of the thigh were due to overlying dressing and clothing.

She progressively improved and reached full enteral feeds. As i.v. access was no longer required, it was decided to remove the catheter at 28 days after insertion. A few attempts by the neonatal and surgical teams to remove the catheter proved futile, at which time the possibility of knot formation in the line was considered. Imaging by X-ray was performed. The X-ray confirmed our suspicion of a knot approximately 2.5 cm from the tip of the PICC (Figure 2).

**Figure 3.**
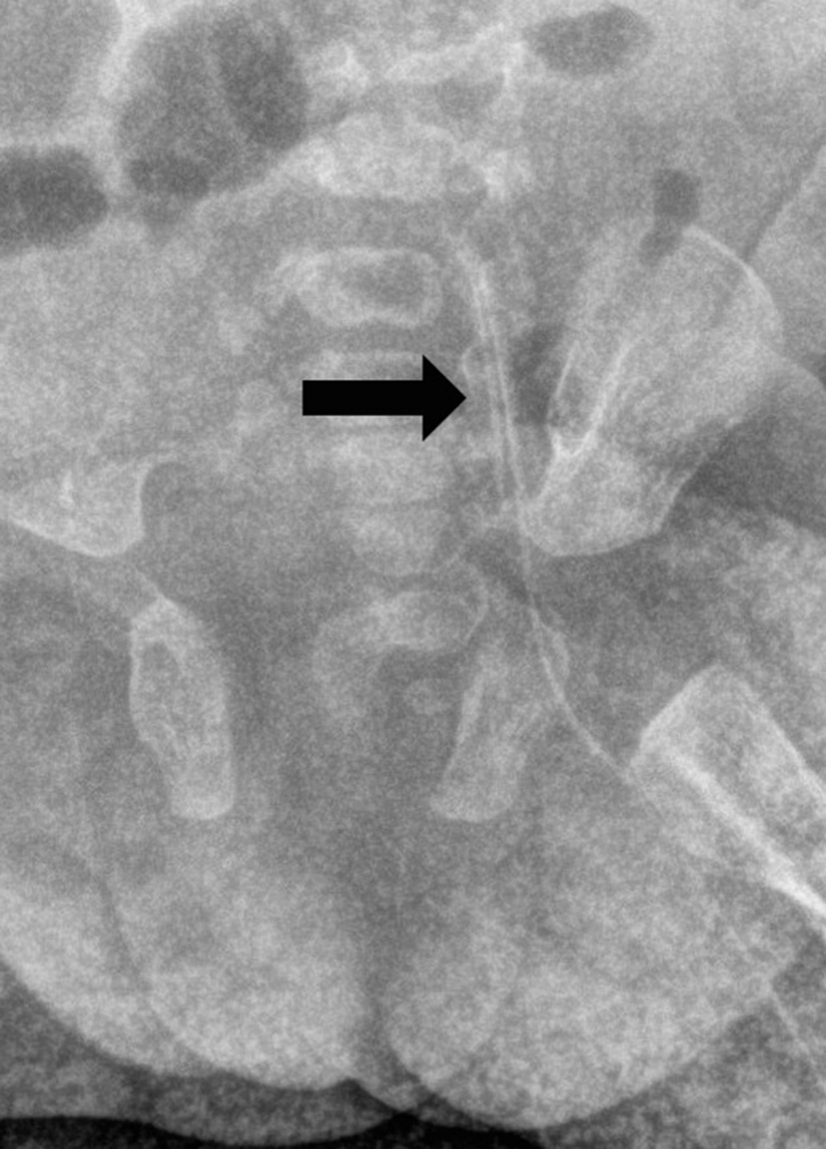
Magnified view of the pelvis from the abdominal plain radiograph demonstrated in-folding of the peripherally inserted central venous catheter (arrow) at the left common and external iliac veins, which was probably the cause of the knot formation.

When resistance was felt upon removal attempt, an earlier abdominal X-ray demonstrated folding of the distal end of the PICC upon itself in the left common iliac vein (Figure 3). However, the significance of this finding was not appreciated by the resident doctor, leading to further attempts at removing the line without imaging guidance, which led to subsequent knot formation and tightening.

The paediatric surgeon felt that the resistance encountered during the PICC retrieval was probably the result of the knot in the catheter getting caught in a valve of the small neonatal long saphenous vein in the upper thigh and venotomy at the entry site of the PICC would not be helpful. After a multidisciplinary discussion between the neonatal, surgical and interventional radiology teams, it was decided that the safest way to remove the catheter was by interventional radiology.

The neonatal long saphenous vein in this prematurely born neonate was very small. An attempt at using a sheath to go over the PICC through the entry site for retrieval could possibly damage and tear the vein. The knot in the PICC appeared tight on X-ray, such that it was very unlikely it could be straightened by a small wire. There was a risk that the PICC could be fractured by the wire.

**Figure 4.**
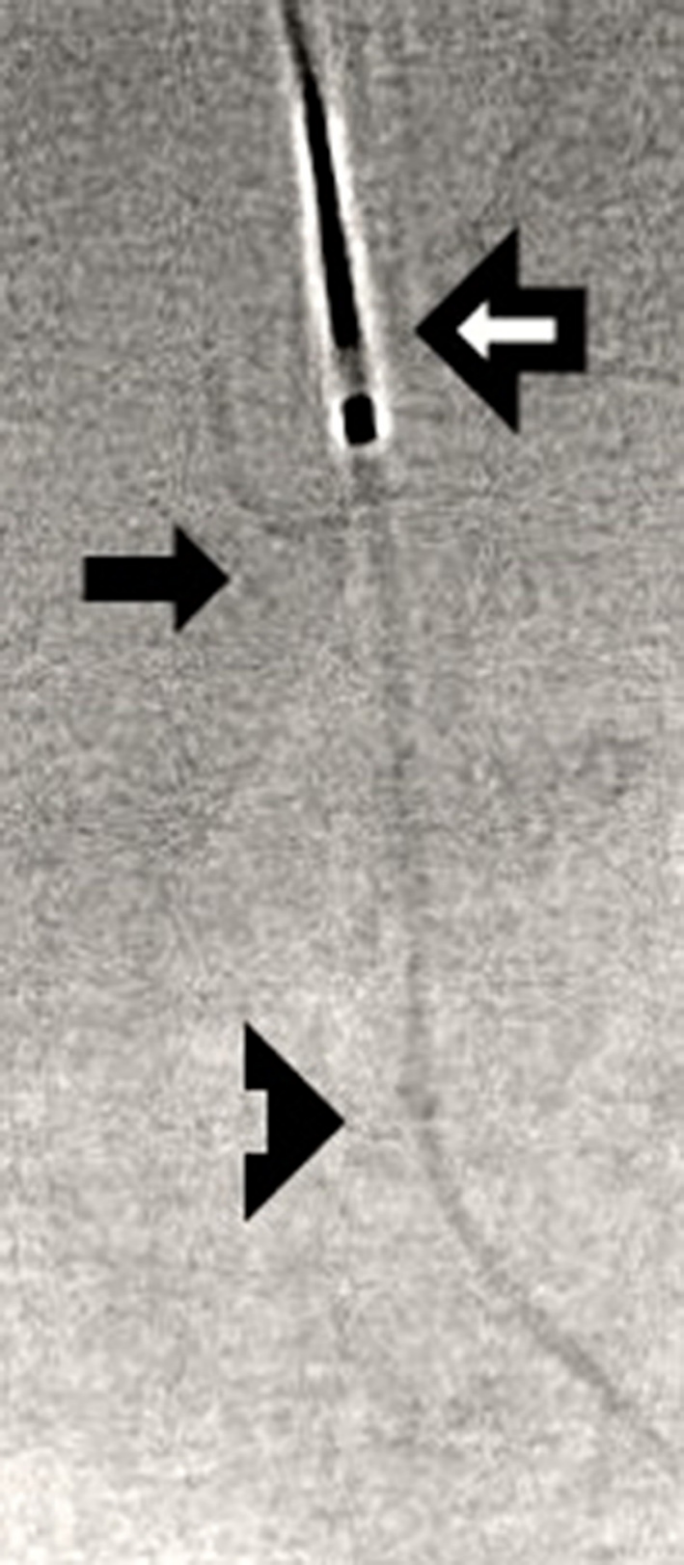
Image captured from low radiation dose fluoroscopy during transjugular retrieval of PICC demonstrated that the microsnare (outlined arrow) secured the distal peripherally inserted central venous catheter (PICC) into a microcatheter (solid arrow ) in the left external iliac vein. The knot of the PICC was seen distally outside the microcatheter (triangular arrow).

**Figure 5.**
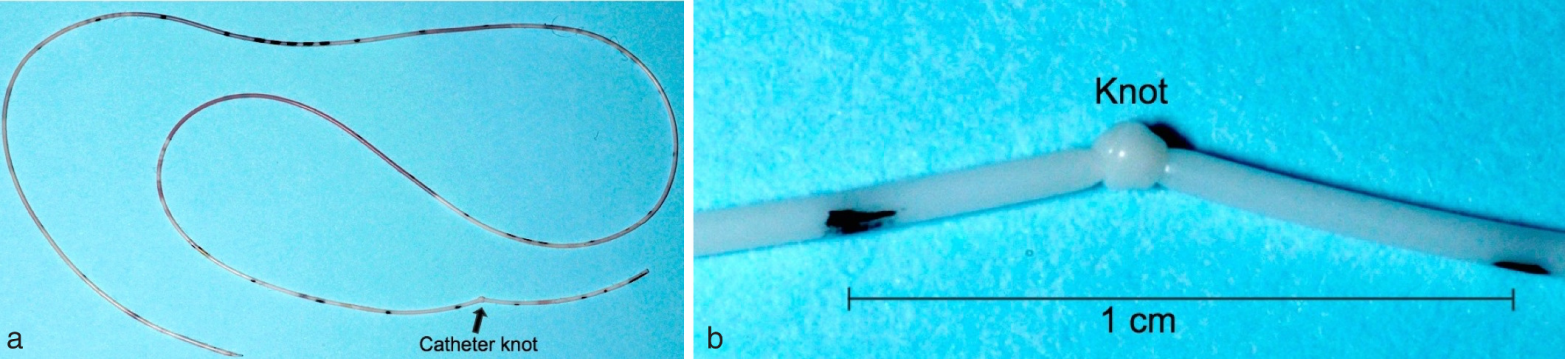
(a) The peripherally inserted central venous catheter (PICC) with knot (arrow). (b) Magnified image of the PICC with knot.

Following informed consent, the neonate was scheduled for a transjugular retrieval of the PICC under general anaesthesia. The procedure was performed under aseptic technique and the exposed PICC was sterilized with aqueous chlorhexidine. The right internal jugular vein was punctured under ultrasound guidance and a 3 French sheath was inserted. An Amplatz GooseNeck Microsnare set with a 2 mm loop (Covidien, Minneapolis, MN) was used. The microsnare reached the left external iliac vein. The tip of the PICC was successfully snared with no difficulty (Figure 4). The PICC was pulled into a microcatheter. Once the knot was pulled back to the level of the right internal jugular vein, the PICC was then cut at its entry site in mid-calf. The remaining PICC was retrieved through the jugular sheath uneventfully (Figure 5a,b).

## Discussion

Knotting of an intravascular catheter was first reported by Johansson et al^3^ in 1954 in the context of cardiac catheterization. Since then, knot formations have been described with other intravascular devices such as pulmonary artery catheters, arteriography catheters, guidewires, pacemaker electrodes and also in PICCs. The factors predisposing to knot formation of the intravascular catheters can be divided into patient factors and/or catheter characteristics.^4^

The soft and flexible nature of most intravascular catheters combined with long lengths of intravascular positioning predisposes the catheter to coil inside the veins. Coiling is often the first step towards knot formation.^4^

The proposed mechanisms of knot formation in PICCs described in the literature include coiling of the soft catheter within veins when negotiating acute angles at the venous junctions, and rarely, slippage of the cannula outside the vein leading to extravascular knotting.^5^ Coiling or knot formation in a PICC should be suspected if undue resistance is felt during line insertion and also during catheter removal. In our case, the mechanism of knot formation during removal of the catheter could not be entirely explained. The softness of the 2 French catheter and the venous valve might have contributed towards the folding of the PICC, which resulted in knotting during further attempts at removal. Failure to recognize the consequence of this led to further traction on the catheter and hence the formation of a tight knot in the catheter. As the tip of the PICC appeared to be in the inferior aspect of the right atrium in this patient on the initial chest radiograph, cardiac pulsation might partly have contributed towards the folding and knotting.

During catheter removal, gentle sustained traction is usually applied to prevent catheter fracture. The neonatal Epicutaneo-Cava catheter (PICC) is a radiopaque silicone tube with an ability to stretch and is particularly flexible. In 2006 Chiang et al^6^ reported a serious complication of PICC rupture in a neonate that led to embolization of the distal fragment in the right atrium.

If difficulty is encountered during removal, plain radiographs are invaluable in confirming or excluding the presence of a knot in a PICC. Junior doctors in the neonatal unit need to be aware of this rare complication to prevent catheter fracture, leading to intracardiac embolization. Careful scrutiny of the plain radiograph in conjunction with a radiologist will prevent any delay in diagnosis. If a knot is detected, it is advisable that both interventional radiologist and paediatric surgeon are consulted so that the most appropriate retrieval method can be established before any further attempts at removal are made. Ultrasound imaging can also play a role in diagnosing knot formation in PICC in limbs.

There are surgical and non-surgical methods of retrieving knotted intravascular catheters. The non-surgical approach consists of interventional radiological techniques such as untying an open knot and removing or goose neck snare retrieval of a closed knot *via* a suitable venous access.^7^

In our case, the knot was very tight. The 2 French 24-gauge silastic catheter was too small to accommodate a standard guidewire to undo a tight knot. The 0.01 fine wire might not be stiff enough to loosen the knot. The surgical exploration of the common iliac vein in the neonate was fraught with significant morbidity; in particular, the knot might have been caught by a venous valve in the small long saphenous vein. Following discussion with the interventional radiologist, it was decided that right transjugular retrieval of the knotted PICC line was the most appropriate and less morbid procedure for our neonate.

## Conclusions

Although it is a rare occurrence, knot formation in a PICC line should be suspected where resistance during line removal is encountered. The role of a plain radiograph in the confirmation of this very rare complication cannot be overemphasized. The method of retrieval depends on knot size, location, patient size, any unusual anatomical variations and available expertise. Interventional radiological retrieval is the preferred option, unless in exceptional circumstances.

## Learning points

1. Knotting of peripherally inserted central venous lines is a rare but potentially serious complication in neonates.
2. If resistance is felt during removal, the possibility of a knot should be considered and investigated to avoid line fragment embolization. Plain radiograph is the imaging modality of choice for identifying a knotted PICC.
3. We suggest the use of interventional radiology to retrieve knotted catheters if the knot cannot be easily accessed and removed.

## Consent

Informed consent for publication of this de-identified case report was gained from the patient's parents.
